# Involvement of Girdin in the Determination of Cell Polarity during Cell Migration

**DOI:** 10.1371/journal.pone.0036681

**Published:** 2012-05-04

**Authors:** Kei Ohara, Atsushi Enomoto, Takuya Kato, Takahiko Hashimoto, Mayu Isotani-Sakakibara, Naoya Asai, Maki Ishida-Takagishi, Liang Weng, Masanori Nakayama, Takashi Watanabe, Katsuhiro Kato, Kozo Kaibuchi, Yoshiki Murakumo, Yoshiki Hirooka, Hidemi Goto, Masahide Takahashi

**Affiliations:** 1 Department of Pathology, Nagoya University Graduate School of Medicine, Nagoya, Japan; 2 Department of Gastroenterology, Nagoya University Graduate School of Medicine, Nagoya, Japan; 3 Division of Molecular Pathology, Center for Neurological Disease and Cancer, Nagoya University Graduate School of Medicine, Nagoya, Japan; 4 Department of Cell Pharmacology, Nagoya University Graduate School of Medicine, Nagoya, Japan; 5 Core Research for Evolutionary Science and Technology (CREST), Japan Science and Technology Agency, Saitama, Japan; 6 Department of Endoscopy, Nagoya University Hospital, Nagoya, Japan; University of Illinois at Chicago, United States of America

## Abstract

Cell migration is a critical cellular process that determines embryonic development and the progression of human diseases. Therefore, cell- or context-specific mechanisms by which multiple promigratory proteins differentially regulate cell migration must be analyzed in detail. Girdin (girders of actin filaments) (also termed GIV, Gα-interacting vesicle associated protein) is an actin-binding protein that regulates migration of various cells such as endothelial cells, smooth muscle cells, neuroblasts, and cancer cells. Here we show that Girdin regulates the establishment of cell polarity, the deregulation of which may result in the disruption of directional cell migration. We found that Girdin interacts with Par-3, a scaffolding protein that is a component of the Par protein complex that has an established role in determining cell polarity. RNA interference-mediated depletion of Girdin leads to impaired polarization of fibroblasts and mammary epithelial cells in a way similar to that observed in Par-3-depleted cells. Accordingly, the expression of Par-3 mutants unable to interact with Girdin abrogates cell polarization in fibroblasts. Further biochemical analysis suggests that Girdin is present in the Par protein complex that includes Par-3, Par-6, and atypical protein kinase C. Considering previous reports showing the role of Girdin in the directional migration of neuroblasts, network formation of endothelial cells, and cancer invasion, these data may provide a specific mechanism by which Girdin regulates cell movement in biological contexts that require directional cell movement.

## Introduction

Previous work has identified many proteins that positively regulate cell migration. This is an area of interest in many fields of research, including development, inflammation, and human diseases including vascular disease and cancer [1]–[5]. These pro-migratory proteins are the regulators of nuclear transcription, intracellular signal transduction, rearrangements of the cytoskeleton (including actin filaments and microtubules), cell adhesion, and intracellular trafficking. Amid growing evidence for various mechanisms involved in the control of migration in many types of cells, one might not expect profound differences between these cells in their capacities and timing for selective use of the molecular tools and mechanisms.

Girders of actin filaments (Girdin), also termed Gα-interacting vesicle associated protein (GIV) is an interesting actin-binding protein [6] which is expressed in limited types of cells including immature endothelial cells [7], immature neuroblasts [8], [9], smooth muscle cells [10], breast and colon cancer cells [11]–[13], and glioblastoma cells [14]. Girdin binds to the actin cytoskeleton as well as many components of intracellular signaling pathways including the serine/threonine kinase Akt/PKB [6], [15], the trimeric G proteins Gαi/s that mediate signaling evoked by G protein–coupled receptors (GPCRs) [16]–[20], epidermal growth factor receptor (EGFR) [21], and *DISC1* (Disrupted-In-Schizophrenia 1), a candidate gene for the development of schizophrenia and major mental disorders [8]. Using animal models and cultured cells, previous studies successfully demonstrated that synergistic interactions between Girdin and its interacting proteins control cell migration that is dependent upon extracellular signals and the local environment. In addition, it is noteworthy that Girdin-deficient mice survive embryogenesis but have defects in postnatal angiogenesis [7] and adult neurogenesis [8], [9], indicating that Girdin's function may be particularly important for migratory events that take place in postnatal and adult periods rather than in the embryonic period [12]. At present, however, it is unclear how Girdin operates in cell migration, how Girdin confers functional specificity to different types of cells, and which aspects of cell motility are regulated by Girdin during postnatal/adult periods.

**Figure 1:**
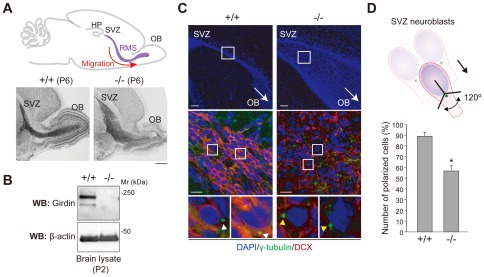
Defects in cell polarity of neuroblasts migrating through the RMS in Girdin^−/−^ mice. A. Upper panel. Schematic illustration of the pathway taken by neuroblasts born in the SVZ that migrate to the OB through the RMS. Lower panels. Nissl-stained brain sections of wild-type (left) and Girdin^−/−^ (right) P6 mice show significant deficits in the chain migration of neuroblasts through the RMS toward the OB in Girdin^−/−^ mice. SVZ, subventricular zone; HP, hippocampus; RMS, rostral migratory stream; OB, olfactory bulb. Scale bar, 500 µm. B. Lysates prepared from the brains of wild-type and and Girdin^−/−^ P2 mice were subjected to Western blot analysis using anti-Girdin antibody. Mr, molecular mass. C, D. Defects in cell polarity of SVZ neuroblasts in Girdin^−/−^ mice. Sagittal sections through the RMS of wild-type (left panels) and Girdin^−/−^ (right panels) P15 mice were stained with anti-Dcx (a marker of immature neuroblasts) (red) and anti-γ-tubulin (green) antibodies. MTOC were frequently found in discrete positions in Girdin^−/−^ mice (yellow arrowheads) in contrast to wild-type mice (white arrowheads), in which most of the MTOC were found in front of the nuclei. Scale bars, 100 µm (upper panels); 20 µm (middle panels). Shown in D is a schematic illustration of the scoring principle: cells with the MTOC located within one third of the cytoplasm relative to the tangential direction were counted. An arrow indicates the direction of the migratory stream. The graph shows the percentage of polarized neuroblasts in wild-type and Girdin^−/−^ mice. A minimum of 40–50 neuroblasts of randomly picked sections from each animal and at least 200 neuroblasts were analyzed for each group. An asterisk indicates statistical significance (Student's t test; P<0.05).

An observation that provides a potential explanation for how Girdin controls cell migration has come from our recent report [9]. It analyzed cohort (or chain) migration of immature neuroblasts born in the subventricular zone (SVZ) toward the olfactory bulb (OB), termed the rostral migratory stream (RMS). Data revealed that migration was severely impaired in the postnatal brains of Girdin-deficient mice [9], [22] (see also Figure 1A). In these mice, SVZ neuroblasts migrated individually and sometimes perpendicular to the direction of the migratory stream, which is in contrast to wild-type littermates in which SVZ neuroblasts form densely-packed, chain-like structures as they migrate in groups within the RMS. These data suggest that cell polarization of neuroblasts may be compromised in Girdin-deficient mice, which results in defective directional chain migration and a widely dispersed and disorganized RMS [9]. However, the molecular mechanisms underlying Girdin's participation in cell polarization have not been defined by previous studies.

Cell polarization is a basic feature of epithelial cells as well as a prerequisite for effective directional cell migration, growth, division, and differentiation. Polarization involves many different proteins functioning in concert, including the regulators of growth factors, chemokine-mediated intracellular signaling pathways, cell adhesion, and arrangement of the cytoskeletons [1], [23]–[28]. Of the various proteins involved in the determination of cell polarity, the most thoroughly characterized are the components of the partitioning-defective (Par) protein complex, which has a conserved function in establishing cell polarity in *C. elegans*, *Drosophila*, and mammals [26], [27], [29], [30]. The Par protein complex is composed of atypical protein kinase C (aPKC) and the conserved PSD-95/Discs-Large/ZO-1 (PDZ) domain-containing proteins, Par-3 (also termed aPKC isotype-specific interacting protein, ASIP) and Par-6. These components play critical roles in epithelium-specific junctional structures, axon-dendrite specification in neurons, and directional cell migration of many types of cells including fibroblasts, astrocytes, and cancer cells [26], [27], [29], [30]–[36]. Targeted deletions of components of the Par protein complex result in embryonic lethal mutations, indicating the crucial roles of these proteins in early development [37]–[40]. In some types of cancers, aberrant inactivation or overexpression of the components of the Par protein complex is closely associated with the development and progression of human cancers [41]–[44]. Although the components of the Par protein complex are involved in multiple processes in many types of cells, there is only partial understanding of how the specificity of the proteins' functions is regulated in specific cell types.

A previous proteomic screen using lysates from HEK293 cells identified several peptides derived from human Girdin in Par-3 immunoprecipitates [45]. In this study, we demonstrate that Girdin biochemically interacts with Par-3, and other components of the Par protein complex. Accordingly, downregulation of Girdin and Par-3 expression and reduced interaction of Girdin/Par-3 resulted in the deregulation of cell polarization of migrating fibroblasts and acinar formation of mammary epithelial cells. Considering the expression patterns of Girdin in certain (but not all) types of cells *in vivo*, Girdin may confer specific function to Par-3 in a context/cell type-specific manner.

## Results

### Girdin-deficient mice demonstrate the loss of polarization of SVZ neuroblasts

We and others previously showed that RNA interference (RNAi)-mediated depletion (knock-down) or targeted deletion of Girdin resulted in defective cell migration of immortalized fibroblasts, endothelial cells, smooth muscle cells, neuroblasts, and breast cancer cells [6], [7], [9], [10], [11], [14], [17], [19]. Among these studies, a clue to the mechanism by which Girdin regulates cell migration has come from our recent observation that Girdin-deficient mice exhibit a severe defect in the migration of neuroblasts with persistent directionality in the RMS, which is a model of collective migration in stream [9], [22] (Figs. 1A, 1B). We immunostained SVZ neuroblasts, which migrate in the RMS from the SVZ to the OB, with γ-tubulin antibody which detects the microtubule organizing center (MTOC), the positioning of which is a hallmark of cell polarity of migrating cells (Figs. 1C, 1D). When cell polarization was assessed based on the positioning of the MTOC relative to the tangential direction (Fig. 1D), we found that the polarization of SVZ neuroblasts in Girdin-deficient mice was significantly disrupted, in contrast to wild-type littermates, in which most of the MTOCs were reoriented in the direction of migration. In Girdin-deficient mice, the random orientation of centrosomes in SVZ neuroblasts suggests that they failed to migrate to the OB (Fig. 1C). It remains possible that intracellular signaling pathways, especially those involved in Akt activation [15], [18] (mediated by chemoattractants/growth factors and/or the microenvironment of migrating neuroblasts) are affected in Girdin-deficient mice. Nonetheless, the data showed potential involvement of Girdin in the determination of cell polarity *in vivo*.

### Girdin interacts with Par-3

**Figure 2:**
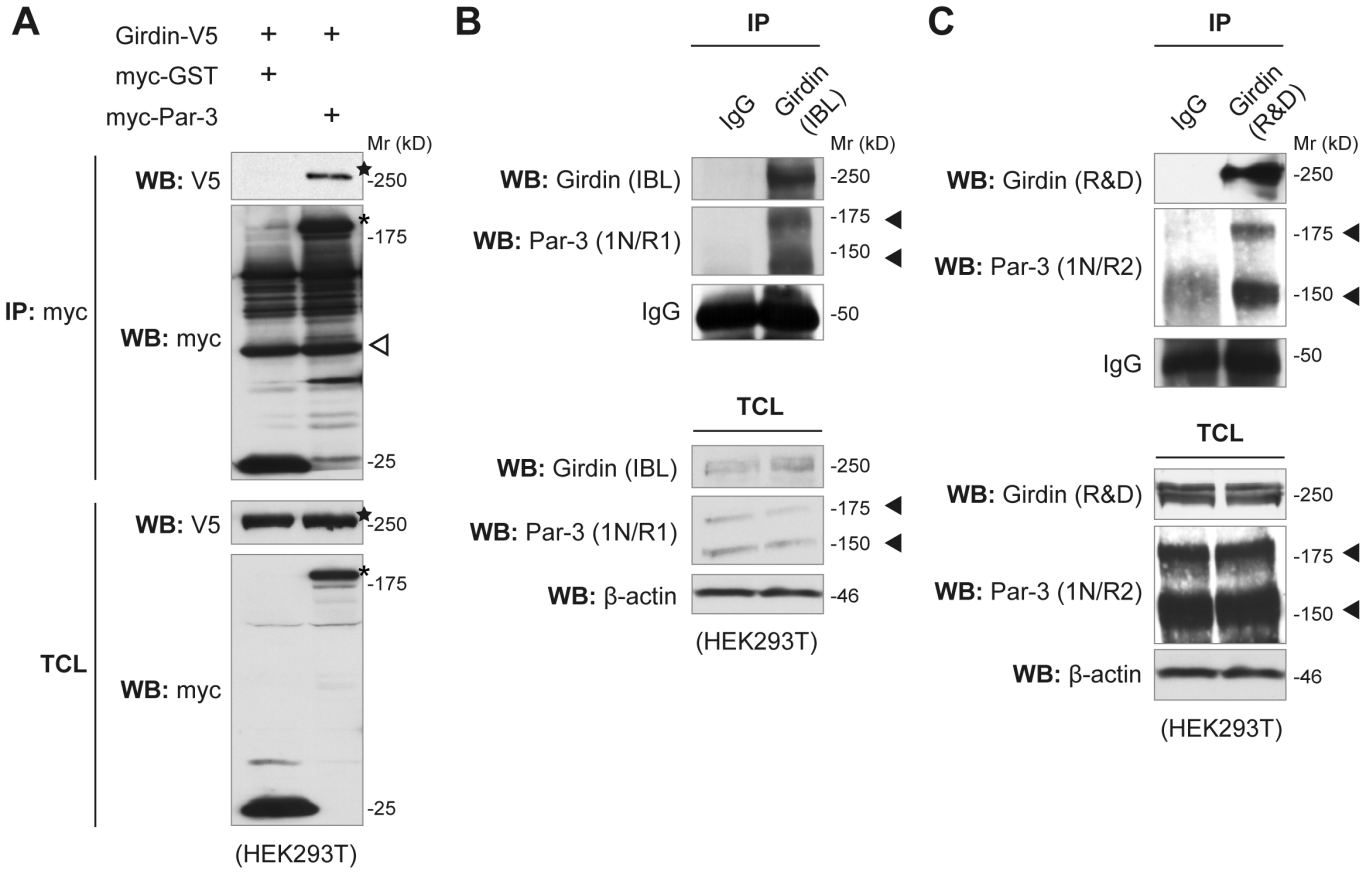
Interaction of Girdin with Par-3. A. Interaction of exogenously expressed Girdin and Par-3 in HEK293T cells. Total cell lysates (TCL) from HEK293T cells that expressed Girdin-V5 and either myc-GST or myc-Par-3 were immunoprecipitated (IP) with anti-myc antibody, followed by Western blot analyses using the indicated antibodies. myc-Par-3 and Girdin-V5 are indicated by asterisks and stars, respectively. Open arrowhead indicates IgG heavy chain. B, C. Interaction between endogenous Girdin and Par-3. Lysates from HEK293T cells were immunoprecipitated with two anti-Girdin antibodies (B, available from IBL; C, available from R&D Systems) and normal rabbit IgG antibodies, followed by Western blot analysis using the indicated antibodies. Two spliced isoforms of Par-3 are indicated by arrowheads.

Investigating the mechanisms by which Girdin regulates cell polarization, we found a previous study in which the techniques of tandem affinity purification combined with liquid chromatography/tandem mass spectrometry identified Girdin as a candidate protein that interacted with Par-3 [45]. In that study, several peptides derived from human Girdin (formerly designated KIAA1212) were identified in trypsin-digestion products. Those proteins were specifically captured in Par-3 immunoprecipitates collected from the extracts of HEK293 human embryonic kidney cells expressing exogenous Par-3. Validation of Girdin as a Par-3 interactant was obtained by our coimmunoprecipitation experiments using extracts from HEK293T cells overexpressing exogenous Girdin and Par-3 (Fig. 2A). We confirmed the interaction of endogenous Girdin and Par-3 by immunoprecipitation experiments using several available antibodies raised against Girdin and Par-3 (Figs. 2B and 2C).

### Mapping Girdin and Par-3 interacting domains

**Figure 3:**
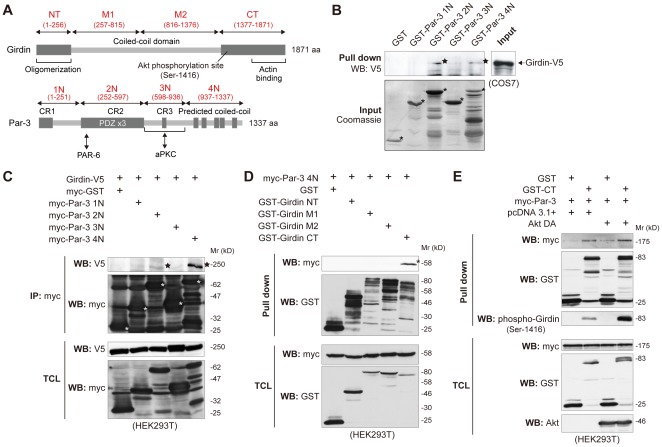
Mapping of interacting domains of Girdin and Par-3. A. Domain structures of human Girdin and Par-3. Fragments and domains of Girdin (upper panel) and Par-3 (lower panel) used in this study are shown. The corresponding amino acid numbers for the fragments and domains are given in parentheses. B. Girdin-binding sites map to the 2N and the 4N domains of Par-3. Lysates from COS7 cells transfected with Girdin-V5 were incubated with purified recombinant Par-3 fragments fused with GST (300 pmol) for 1 hr at 4°C, followed by precipitation with glutathione-Sepharose beads. Coomassie brilliant blue staining (lower panel) shows the input GST-fusion proteins (asterisks) used in the assay. Girdin-V5 that bound to GST-Par-3 2N and 4N is indicated by stars. C. HEK293T cells were transfected with Girdin-V5 and each domain of Par-3, fused to a myc epitope at the N-terminus. Fourty-eight hrs after transfection, the cell were lysed and immunoprecipitated with anti-myc antibody. Immunoprecipitated Par-3 fragments and bound Girdin are indicated by white asterisks and black stars, respectively. D. Requirement of the Girdin CT domain for interaction with Par-3. Lysates from HEK293T cells transfected with the myc-Par-3 4N domain and each domain of Girdin, fused to GST tag at the N-terminus, were precipitated with glutathione-Sepharose beads. The precipitates and the total cell lysates were analysed by Western blot using the indicated antibodies. An asterisk indicates myc-Par-3 4N that bound to GST-Girdin CT. E. No apparent effects of Girdin phosphorylation on its interaction with Par-3. HEK293T cells were transfected with the indicated combination of myc-Par-3, GST, Girdin CT domain fused with GST (GST-CT), and a dominant active form of Akt (Akt DA). Fourty-eight hrs after transfection, the lysates were precipitated with glutathione-Sepharose beads and eluted proteins were analysed by the indicated antibodies.

Par-3 consists of multiple domains that determine its function and provide the interfaces for interaction with other proteins and is considered to be a scaffold protein that coordinates the intracellular locations of its interacting proteins (Fig. 3A) [27], [29]. Previous studies have identified multiple proteins that interact with Par-3, including Par-6, aPKC, KIF3A (a member of the kinesin microtubule-associated motor protein family), T-lymphoma invasion and metastasis 1 (Tiam1), and Rho-kinase [27], [29], [30], [46]–[49]. To identify which domains of Girdin and Par-3 were responsible for their interaction, we performed *in vitro* binding assays using purified recombinant Par-3 proteins and immunoprecipitation experiments using lysates extracted from COS7 or HEK293T cells overexpressing fragments of each protein (Figs. 3B, 3C). We found that both 2N and 4N domains (the PDZ binding domain and the coiled-coil domain, respectively) of Par-3 have the potential to bind Girdin, implying that the mode of the interaction is complex and conceivably influenced by indirect mechanisms, as was also discussed in the studies of the interactions of Par-3/Tiam1 and Par-3/Tiam2 (also termed STEF, SIF and Tiam 1-like exchange factor) [47]. Although the data open up many possibilities, it is of note that the interaction between the 4N domain of Par-3 and Girdin was quite evident (Fig. 3C) and consistently detectable in a series of experiments, providing the basis for the following experiments, in which we focused on the interaction between the 4N domain of Par-3 and Girdin.

Girdin possesses a long coiled-coil domain that spans more than two-thirds of the middle of the protein, which is flanked by unique amino- (NT) and carboxy- (CT) terminal actin-binding domains (Fig. 3A) [6], [12], [50]. Our glutathione S-transferase (GST)-pull down assays using the lysates from cells expressing Par-3 4N domain and each domain of Girdin showed that the Girdin CT domain is responsible for the interaction with Par-3 (Fig. 3D). Our attempts with smaller constructs to narrow down the domains/regions of Girdin for Par-3 interaction were unsuccessful, suggesting that the entire Girdin CT domain may be necessary for Par-3 interaction (data not shown).

Since we previously identified Girdin as a direct target for Akt phosphorylation [6], which is important for the migration of endothelial and cancer cells [7], [11], we asked whether Akt-mediated phosphorylation of Girdin at the serine-1416 site affected its interaction with Par-3 (Fig. 3E). Overexpression of a dominant-active form of Akt (Akt DA) effectively induced Girdin phosphorylation as assessed by Western blot analysis using anti-phospho-Girdin antibody. However, it had no apparent effects on the interaction between the Girdin CT domain and Par-3. The result indicates that Akt, which is activated downstream from various receptor tyrosine kinases, may not be employed to regulate Girdin/Par-3 interaction in the experimental conditions used here.

### Direct interaction of Girdin with Par-3

**Figure 4:**
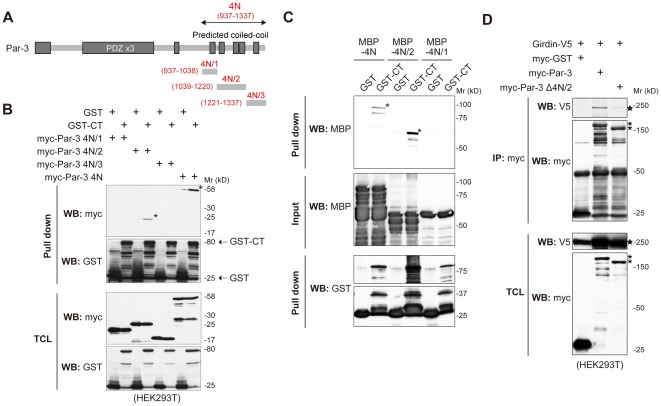
Identifying the Par-3 domain responsible for the interaction with Girdin. A. Fragments of the Par-3 4N domain used in the study are shown. The 4N domain of Par-3 was further divided into three subdomains (4N/1, 4N/2, and 4N/3). The corresponding amino acid numbers for the fragments are indicated in parenthesis. B. HEK293T cells were transfected with the indicated combination of each subdomain of myc-Par-3 4N, GST, and GST-CT. Lysates were precipitated with glutathione-Sepharose beads and eluted proteins were analysed with the indicated antibodies. Par-3 4N and 4N/2 domains that bound to GST-CT are indicated by asterisks. C. Direct interaction between the 4N/2 region of Par-3 and Girdin. Purified recombinant MBP (maltose binding protein)-fusion proteins containing the 4N, 4N/2, and 4N/1 regions of Par-3 (300 pmol) were incubated with 50 pmol of recombinant GST of GST-CT conjugated with glutathione beads for 1 hr at 4°C, washed three times, eluted with 10 mM reduced glutathione, separated on SDS-polyacrylamide gels, and subjected to Western blot analyses using the indicated antibodies. Asterisks, bound MBP-fusion proteins. White asterisks, MBP-fusion protein input. D. Total cell lysates from HEK293T cells that expressed Girdin-V5 and either myc-GST, myc-Par-3, or myc-Par-3 Δ4N/2 were immunoprecipitated with anti-myc antibody, followed by Western blot analyses using the indicated antibodies. myc-Par-3 (wild type and Δ4N/2) and Girdin-V5 are indicated by asterisks and stars, respectively.

Next, considering that the Par-3 4N domain provides interfaces to bind several important proteins that determine the function of Par-3, we narrowed down the domain of Par-3 that interacts with Girdin (Figs. 4A, B). The result showed that the CT domain of Girdin interacts with the middle part of the 4N domain (termed 4N/2) of Par-3, which has also been reported to be responsible for interaction with KIF3A [46].

Given previous studies showing that numerous proteins interact with Par-3, it was important to determine the mode of interaction between the two proteins. Our GST pull-down assays using purified recombinant proteins of Par-3 fragments and the Girdin CT domain showed a direct interaction of Girdin with the 4N/2, but not the 4N/1 (control) fragment of Par-3 *in vitro* (Fig. 4C). Consistent with this was the observation that a Par-3 mutant (Δ4N/2) lost the ability to interact with Girdin, indicating that the 4N/2 region is the most important domain in binding to Girdin (Fig. 4D). However, it is of note that we observed a faint interaction between the Par-3 mutant Δ4N/2 and Girdin, suggesting the possibility that Par-3 interacts with Girdin via other domains (presumably the 2N domain) or the Δ4N/2 mutant indirectly interacts with Girdin in cells.

### Girdin interacts with Par-3 at the cell periphery and in the cytoplasm in migrating cells

**Figure 5:**
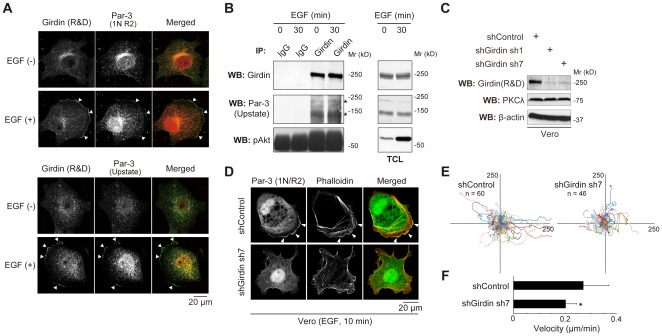
Colocalization of Girdin with Par-3 and the involvement of Girdin in random haptotoctic migration. A. Colocalization of Girdin with Par-3 in Vero cells. Quiescent (upper panels) and EGF-stimulated (EGF (+), lower panels) Vero cells were fixed and stained with anti-Girdin (green) and anti-Par-3 (red) antibodies, showing the colocalization of both proteins at the leading edge of migrating cells. Colocalization between the two proteins was shown by immunocytochemistry using two available Par-3 antibodies (upper panel, Par-3 antibody generated by us previously; lower panel, commercially available Par-3 antibody). Arrowheads indicate the localization of the two proteins at the leading edge. B. Interaction of endogenous Girdin with Par-3 in Vero cells. Lysates from Vero cells, either stimulated for 30 min or non-stimulated with EGF (100 ng/mL), were immunoprecipitated with anti-Girdin (IBL) and normal rabbit IgG antibodies, followed by Western blot analysis using the indicated antibodies. Two spliced isoforms of Par-3 are indicated by asterisks. C. Analysis of the efficiency of retrovirus-delivered shRNAs in silencing endogenous Girdin in Vero cells. Vero cells were infected with retroviruses expressing control or Girdin shRNAs. Cell lysates were collected five days post-infection and analysed by Western blot analysis using the indicated antibodies. D. Effects of Girdin depletion on Par-3 localization in Vero cells. Control- or Girdin shRNA-expressing Vero cells stimulated with EGF for ten min were fixed and stained with Par-3 antibody (green) and Alexa 594-conjugated phalloidin (red). Arrowheads indicate lamellipodia at the leading edge. E, F. Tracks of control and Girdin-depleted Vero cells on collagen type I-coated glass based dishes (E). Paths of the nuclei of cells are recorded every four min for 16 hr following the methods described in Materials and Methods (n = 60 and 46 for control and Girdin-depleted cells, respectively). Axis labels are in pixels. (F) Migration velocity of control and Girdin-depleted Vero cells. An asterisk indicates significant difference (Student's t test; P<0.001).

We and others previously reported that Girdin localizes with the actin cytoskeleton at the cell periphery as well as diffusely in the cytoplasm [6], [7], [16], [17]. In migrating cells stimulated with extracellular cues including growth factors, Girdin and its phosphorylated form preferentially localize at the leading edge [6], [7]. In the present study, we examined the colocalization of Girdin and Par-3 in Vero cells with or without EGF stimulation (Fig. 5A). In quiescent cells, both proteins were localized in the cytoplasm around the nucleus, whereas in EGF-stimulated cells, the colocalization predominantly took place in the lamellipodia, the actin-rich protruding membrane structures formed at the leading edge (Fig. 5A). We confirmed the colocalization of the two proteins by immunocytostaining procedures using two distinct anti-Par-3 antibodies, one generated and used in previous studies [47], [49] and one commercially available. Immunoprecipitation assays showed that Girdin interacted with Par-3 independently of EGF stimulation (Fig. 5B), suggesting other unknown mechanisms regulating the localization of the Girdin/Par-3 complex at the leading edge.

### Effects of Girdin depletion on cell migration and polarization in Vero cells

Based on the phenotype found in SVZ neuroblasts in Girdin-deficient mice (Fig. 1) and biochemical interaction of Girdin with Par-3 (Figs. 2, 3, and 4), we next asked whether Girdin-depletion disrupted cell migration and polarity. We depleted Girdin by infecting cells with a retrovirus engineered to express Girdin short hairpin RNA (shRNA). We observed changes in the morphology of EGF-stimulated cells, leading to the attenuation of lamellipodia formation and Par-3 localization at the leading edge (Figs. 5C, D). For the assessment of Girdin's function in cell migration and polarization, we set out to test random haptotactic movement and directional movement using *in vitro* experimental models. As with the immunocytochemistry, we used Vero cells, which display morphological changes with a front-rear polarity when stimulated to undergo directional migration [51]. Time-lapse recordings of Vero cells seeded on glass-based dishes showed that the depletion of Girdin resulted in decreases in both the velocity of cells migrating randomly and the distance they migrated (Figs. 5E, F), showing that Girdin is essential for random haptotactic cell migration.

**Figure 6:**
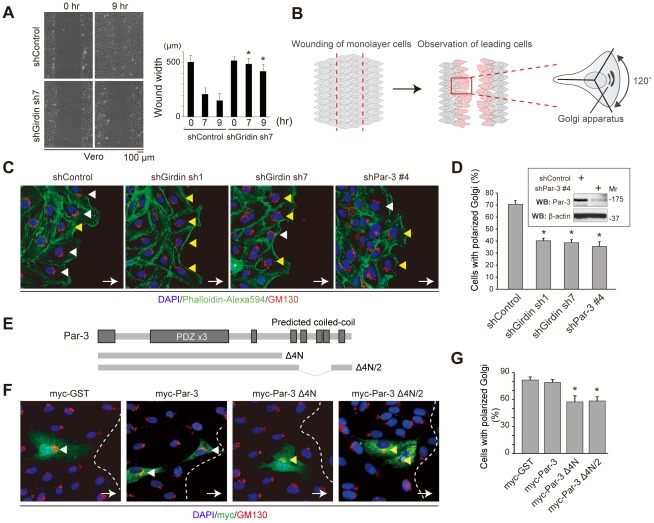
Involvement of Girdin in the establishment of cell polarity. A. Girdin depletion inhibited directional cell migration. Control or Girdin-depleted Vero cells (eight dishes for each group) were scratched to induce cell migration and incubated in medium supplemented with 3% FBS for nine hr. The wound's width was measured in each group at zero, seven, and nine hr after the wounding. Asterisks indicate significant difference compared with control cells (Student's t test; P<0.05). B. Schematic illustration of scoring principle of cell polarity in Vero cells. Monolayers of confluent Vero cells on glass-based dishes were scratched to initiate sheet migration into the wound (left), incubated for eight hrs, fixed, and immunostained for GM130 (a marker of the Golgi apparatus). Polarization of leading cells that face the wound was analyzed by the location of the Golgi apparatus (middle). Cells with the Golgi apparatus locating within one third of the cytoplasm relative to the direction of sheet migration were counted (right). C, D. Depletion of Girdin and Par-3 from Vero cells: effects on cell polarization. Monolayers of control- (far left panel), Girdin- (sh1 and sh7, middle panels), and Par-3 shRNA-expressing Vero cells (far right panel) were scratched to induce migration and the polarization of leading cells was evaluated by immunostaining for GM130 (red). Nuclei and actin filaments were visualized by staining with DAPI (blue) and phalloidin (green), respectively. Properly polarized and non-polarized leading cells are indicated by white and yellow arrowheads, respectively. In D, the graph shows the percentage of leading cells with polarized Golgi apparatus in each group (300 cells from three independent experiments). Inset shows the data from Western blot analysis showing stable depletion of endogenous Par-3 by the infection of a retrovirus expressing Par-3 shRNA. Asterisks indicate statistical significance (Student's t test; P<0.05). E, F, G. Overexpression of Par-3 Δ4N and Δ4N/2 mutants in Vero cells: effects on cell polarization. Shown in E is a schematic illustration of Par-3 Δ4N and Δ4N/2 mutants used in the study. Monolayers of control (myc-GST), Par-3 Δ4N-, and Par-3 Δ4N/2-overexpressing Vero cells were scratched to induce sheet migration. The polarization of leading cells, immunostained for GM130 (F), and the percentage of leading cells with polarized Golgi apparatus was quantified in each group (150 cells each) (G). The asterisk indicates statistical significance (Student's t test; P<0.05).

Next, to determine whether Girdin was involved in determining cell polarity, we examined the role of Girdin in directional cell migration using an *in vitro* wound healing assay model, in which a confluent monolayer of Vero cells was wounded to stimulate their sheet migration (Fig. 6). In Girdin-depleted cells, we found that the closure of the wounded area was significantly slower than that of control cells (Fig. 6A). Immunostaining with an antibody for GM130, a marker for the Golgi apparatus, showed that the location of the Golgi apparatus was reoriented toward the direction of cell migration in leading cells that faced the wound (Figs. 6B–D). Importantly, the depletion of endogenous Girdin resulted in a significant decrease in the number of polarized leading cells, as also observed for Par-3-depleted cells (Figs. 6C, D). In those experiments, we used two different target sequences for Girdin depletion, the efficacy of which was tested in Western blot analysis (Fig. 5C), to rule out off-target effects of the shRNAs.

We next found that the overexpression of mutants of Par-3 (Par-3 Δ4N and Δ4N/2), which seemed to lack the ability to interact with Girdin (Figs. 3, 4, 6E), also had adverse effects on cell polarization (Figs. 6F, G). In wound healing assays, leading cells transfected with Par-3 Δ4N or Δ4N/2 exhibited a significant inhibition in the reorientation of the Golgi apparatus in cells migrating into the wound (Figs. 6F, G). While not necessarily conclusive, the data clearly suggest that Girdin/Par-3 interaction has an important role in cell polarization during cell migration.

### Depletion of Girdin inhibits the formation of acini in 3D cultures of mammary epithelial cells

**Figure 7:**
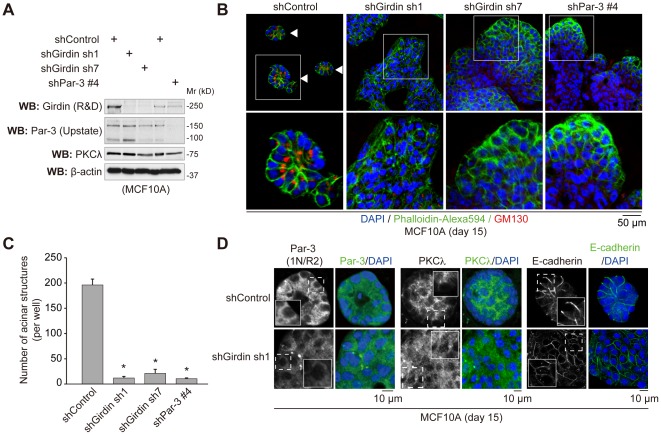
Girdin depletion from MCF10A cells: effects on the formation of polarized acinar structures. A. Analysis of the efficiency of retrovirus-delivered shRNAs in silencing endogenous Girdin and Par-3 in MCF10A cells. MCF10A cells were infected with retroviruses expressing control, Girdin, and Par-3 shRNAs. Cell lysates were collected seven days post-infection and subjected to Western blot analysis using the indicated antibodies. B. Morphology of acinar or sheet structures formed by control, Girdin, and Par-3 shRNA-expressing MCF10A cells plated on Matrigel for 15 days. The acini (arrowheads, far left panel) or sheet structures (middle panel and far left panels) were labeled for actin (green), GM130 (red), and nuclei (blue) and representative confocal images are shown. The regions within white boxes are shown at a higher magnification in lower panels. C. Numbers of acini formed by control, Girdin, and Par-3 shRNA-expressing MCF10A cells in Matrigel were determined on day 15. The experiment was repeated three times. Data are expressed as the mean ± S.E.M., and comparison between control shRNA and Girdin/Par-3 shRNA was done by Student's t-test. Asterisks indicate significant differences (P<0.05). D. Localization of Par-3, aPKC, and E-cadherin in Girdin-depleted MCF10A cells. Control and Girdin shRNA-expressing MCF10A cells plated on Matrigel for 15 days were fixed and stained with the indicated antibodies (green) and DAPI (blue).

In the body, apical-basal cell polarity is a fundamental feature of all epithelial cells to maintain tissue integrity, the loss of which is a hallmark of the most advanced malignant tumors [1], [26], [27], [29], [52], [53]. To examine whether Girdin is involved in the establishment of apical-basal cell polarity, we used an *in vitro* morphogenesis model in which MCF10A cells, a human mammary epithelial cell line, form hollow acinar structures in 3D culture conditions [54]–[57]. We first established MCF10A cells stably expressing either Girdin shRNA (sh1 and sh7) or Par-3 shRNA, in which endogenous Girdin or Par-3, respectively, was effectively depleted (Fig. 7A). We found adverse effects of Girdin depletion on Par-3 expression, and vice versa, leading to the speculation that it is attributable to the toxicity of Girdin or Par-3 depletion or that the expression of Girdin and Par-3 is synergistically regulated. We found that the depletion of Girdin resulted in a similar degree of suppression in the generation of acinar structures as seen for Par-3 (Figs. 7B, C). Notably, in contrast to control MCF10A cells that formed compact acinar structures with a single layer of polarized cells, Girdin-depleted cells, as well as Par-3-depleted cells, proliferated in a sheet-like fashion and exhibited a cobblestone appearance in the Matrigel (Fig. 7B). Although we could not ignore the effect of Girdin or Par-3 depletion on cell proliferation, the attachment to the basement membrane, the synthesis of junctional proteins, and the formation of apical junctional complexes during the process of acinar formation, these data suggest the possibility that Girdin as well as Par-3 participate in the process of polarization of MCF10A cells which is required for morphogenesis in the 3D culture system. Another important issue not resolved in the present study concerns the role of Girdin/Par-3 interaction in the formation of the acinar structure. Immunostaining of E-cadherin, as well as Par-3 and PKCλ (a member of the aPKC family), however, showed no apparent alteration in the distribution of these proteins (Fig. 7D), making it unclear how Girdin regulates the function and subcellular localization of Par-3 and the formation of the acinar structures by MCF10A cells.

### Involvement of Girdin in the Par protein complex

**Figure 8:**
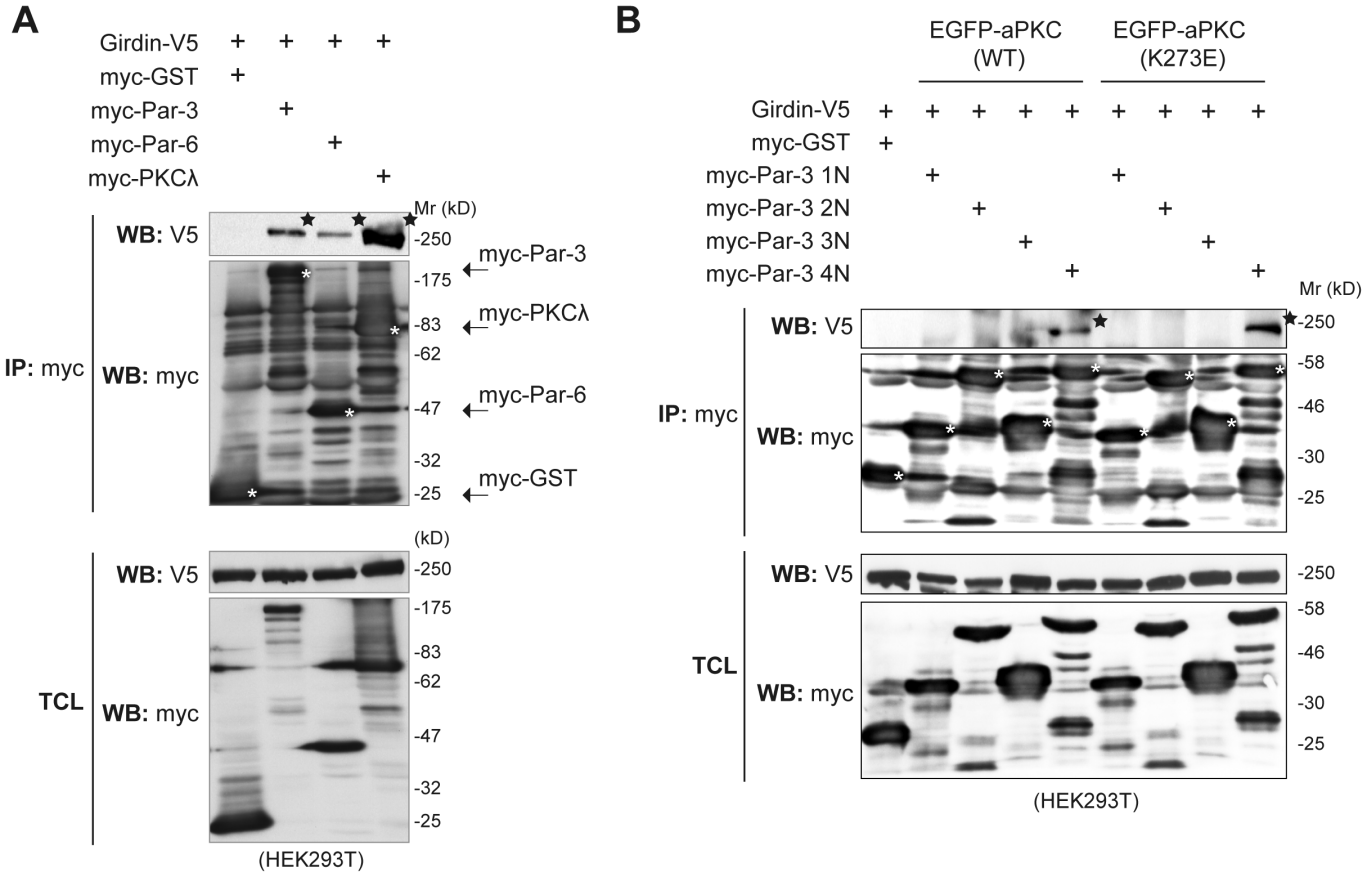
Involvement of Girdin in the Par protein complex. A. Interaction among exogenously expressed Girdin and Par-3, Par-6, and PKCλ in HEK293T cells. Total cell lysates from HEK293T cells expressing Girdin-V5 and either myc-GST, myc-Par-3, myc-Par-6, or myc-PKCλ were immunoprecipitated with anti-myc antibody, followed by Western blot analyses with anti-V5 antibody. Asterisks indicated the immunoprecipitated proteins. Coimmunoprecipitated Girdin-V5 was indicated by stars. B. Girdin interacts with Par-3 independently of the kinase activity of aPKC. Total cell lysates from HEK293T cells expressing the indicated combination of plasmids were immunoprecipitated with anti-myc antibody, followed by Western blot analyses with anti-V5 antibody. White asterisks indicated the immunoprecipitated proteins. Stars indicate coimmunoprecipitated Girdin-V5.

Looking at the effects of Girdin depletion on cell polarization in Vero (Figs. 6C, D) and MCF10A (Figs. 7B, C) cells, an intriguing speculation is that Girdin may function in line with other components of the Par protein complex. Our GST-pull down assays and immunoprecipitation experiments using lysates from cells overexpressing exogenous Par-3, Par-6, and PKCλ revealed that Girdin interacts with a protein complex that includes Par-6 and aPKC (Fig. 8A). Although the mode of interaction between Girdin and the Par protein complex was not unequivocally characterized, our findings implicate functional involvement of Girdin in multiple cellular processes regulated by the Par protein complex. We point out that exogenous expression of a dominant-negative mutant (K273E) of PKC did not affect the interaction of the 4N domain of Par-3 with Girdin (Fig. 8B), suggesting that Girdin/Par-3 interaction is not regulated by the kinase activity of aPKC.

## Discussion

Cell polarization is a fundamental cellular process that governs embryonic development, morphogenesis, repair, and homeostasis of the body, the loss of which results in serious outcomes, including embryonic lethality and cancer development [26], [27], [29]–[36], [53]. Many proteins and protein complexes, including the Par and the Scribble/Discs large (Dlg)/Lethal giant larvae (Lgl) protein complexes [58], [59], are known to be involved in cell polarization. However, these known regulators of cell polarity, generically termed “cell polarity proteins”, are essential for almost all types of cells, as are housekeeping cytoskeletal proteins, making it unclear how these cell polarity proteins determine cellular properties specific for a given situation. Thus, it is imperative to identify more mechanisms mediating the function of the cell polarity proteins in various cellular processes.

Based on proteomic techniques that screened proteins interacting with cell polarity proteins by [45], we here identified Girdin as a novel protein involved in the Par protein complex. Although the precise mechanisms of interaction, including evidence for either direct or indirect interaction between Girdin and the constituents of the Par protein complex, require further delineation, our study has revealed a new aspect of the Par protein complex, at least in regard to its *in vivo* roles in postnatal and adult life as noted below.

We previously reported that Girdin-deficient mice that were generated by targeted gene disruption were viable and showed no apparent gross abnormalities at birth [7], [8]. Those results suggested that Girdin was dispensable for embryonic development, contrasting starkly with Par-3-deficient mice which die at embryonic day 13–15 due to defects in cardiovascular development [37]. It is of particular interest to note that Girdin is essential for the migration of endothelial cells during postnatal angiogenesis that takes place in ocular and brain vascular development [7] and neuronal migration during postnatal/adult neurogenesis that selectively occurs in the hippocampal dentate gyrus and the SVZ [8], [9], [60]. The present study provides a clue how these cells use Girdin and cell polarity proteins to respond to extracellular cues and move with directionality in these biological contexts. Accordingly, we showed that cell polarization of SVZ neuroblasts migrating in the RMS from the SVZ toward the OB was deregulated in Girdin-deficient mice (Fig. 1A) [9]. In addition, we recently reported that neuroblasts born in the DG of the hippocampus exhibit uncontrolled migration, leading to their mispositioning [8]. The future challenge will be to reveal how deregulation of Girdin/Par-3 interaction is involved in such aberrant cellular migration *in vivo*.

Many studies suggest that a primary function of Par-3 is to act as a scaffold or a hub protein that recruits many other proteins [27], [29]. Among them is KIF3A, which has been reported to bind to the 4N/2 region of Par-3 to regulate neuronal polarity [46]. An important but elusive issue is whether Girdin and KIF3A, which share the binding region (4N/2) of Par-3 with Girdin, compete with each other during the migration of neuroblasts and endothelial cells. We previously found that Girdin depletion had no apparent role in axon-dendrite determination [8], in contrast to the marked participation of Par-3/KIF3A interaction [46]. These findings imply that the function of Girdin may not be closely linked to that of KIF3A, reflecting an intricate network of Par-3-interacting proteins. In this regard, our recent finding that the interactions among the components of the Par protein complex are dynamically regulated by protein phosphorylation, must be considered [49]. That observation leads to the hypothesis that components of the Par protein complex are interchangeable, depending on the extracellular signals that cells sense and the biological contexts.

Another clue to the mechanism of Girdin-mediated cell migration and polarization is that cell porality proteins including Par-3 were identified by a genome-wide screen as regulators for the endocytic trafficking pathway [61]. Considering the localization of Girdin molecules on the network of actin filaments just beneath the plasma membrane [7], cell-cell contact sites in neuroblasts [9], and intracellular vesicles [16], it is plausible to speculate that Girdin cooperates with Par-3 to regulate endocytosis at the leading edge or the cell-cell contact sites, which is critical for polarized migration of cells. Also, whether Girdin-mediated regulation of the functions of DISC1 and Gαi/s converges on a common machinery of cell migration and polarization is a matter of further studies.

Reflecting that cancers share mechanistic features with those observed in development, Girdin is aberrantly expressed in certain types of cancer, including breast and colon cancers, and glioblastomas [11], [13], [14]. Other studies have demonstrated that alterations in the expression and the function of cell polarity proteins Par-3, Par-6, and aPKC are related to the progression of human cancers [41]–[44]. Consistent with this is our present study that showed that ectopic overexpression of Par-3 Δ4N or Δ4N/2 mutants resulted in the deregulation of cell polarization during cell migration (Figs. 6F, G). In view of the fact that the most poorly differentiated carcinomas exhibit an absence of glandular differentiation accompanied by a loss of cellular polarity, it will be interesting to investigate in future clinical studies whether the expression levels of Girdin and its associating cell polarity proteins are related to differentiation stages of tumors and patients' prognosis. Also, an experimental challenge is to verify how Girdin and its transcriptional regulation are involved in the determination of cell polarization and tissue architecture in human malignancies.

## Materials and Methods

### Ethics statement

All animal protocols were approved by the Animal Care and Use Committee of Nagoya University Graduate School of Medicine (Approval ID 22342).

### Plasmids and antibodies

The cDNA for rat Par-3 (ASIP) [62] was generously provided by Shigeo Ohno (Yokohama City University, Kanagawa, Japan). The construction of Girdin, Par-3, aPKC (PKCλ), and rat Par-6 expression vectors was previously described [6], [46], [47], [49]. Rabbit anti-Girdin polyclonal antibody was developed against the 19 carboxyl-terminal amino acids of Girdin and affinity-purified with the immunized peptide [6], which is currently commercially available (IBL, Gumma, Japan). Generation of rabbit anti-phosphorylated Girdin and anti-Par-3 polyclonal antibodies (1N/R1 and 1N/R2) was performed as previously described [6], [46], [47], [49]. Other antibodies used in this study included anti-Par-3 rabbit polyclonal antibody (Millipore, Bedford, MA), anti-Girdin sheep polyclonal antibody (R&D Systems, Minneapolis, MN), anti-GM130 rabbit monoclonal antibody (Abcam, Cambridge, UK), anti-GM130 mouse monoclonal antibody (BD Bioscience, San Jose, CA), anti-E-cadherin mouse monoclonal antibody (BD Bioscience), anti-GST monoclonal antibody (Cell Signaling Technology, Danvers, MA), anti-MBP antiserum (New England Biolabs, Beverly, MA), anti-GFP polyclonal antibody (MBL, Nagoya, Japan), anti-β-actin mouse monoclonal antibody (Sigma, St Louis, MO), anti-γ-tubulin mouse monoclonal antibody (Sigma), anti-Dcx (doublecortin) goat polyclonal antibody (Santa Cruz Biotechnology), anti-V5 monoclonal antibody (Invitrogen, Carlsbad, CA), and anti-c-myc monoclonal antibody (Santa Cruz Biotechnology, Santa Cruz, CA).

### Girdin knockout mice

Construction of the Girdin gene-targeting vector and generation of Girdin knockout mice were described previously [7].

### Immunofluorescent study and Nissl staining of the RMS sections

Brains of wild-type and Girdin^−/−^ postnatal day (P)15 mice were perfused with 4% paraformaldehyde in 0.1 M phosphate buffer, postfixed in the same fixative overnight, cut into sections on a microslicer (VT1200S, Leica) and immunolabeled for fluorescence microscopy. For immunofluorescence studies, sections were incubated with anti-γ-tubulin and Dcx antibodies diluted in PBS and applied to sections overnight at 4°C. After washes with phosphate-buffered saline (PBS) (10 mmol/L sodium phosphate, 150 mmol/L NaCl, pH 7.4), sections were incubated with Alexa Fluor 488/594-conjugated secondary antibodies (Invitrogen), diluted in PBS for three hrs at 25°C. The sections were then mounted with ProLong Gold antifade reagent containing DAPI (4′,6-diamidino-2-phenylindole) (Invitrogen), and fluorescence was examined using a confocal laser-scanning microscope (Fluoview FV500; Olympus, Tokyo, Japan). For cresyl violet (Nissl) staining, paraffin-embedded brain tissue sections were deparaffinized and placed in 0.5% cresyl violet in distilled water at 37°C for 10 min. The sections were briefly rinsed twice in 90% ethanol and then dipped in 100% ethanol three times before being dehydrated in xylene three times for five min. Sections were covered with glass coverslips adhering to Permount and examined under a light microscope.

### Culture of cell lines

COS7, Vero, and HEK293T cells, purchased from American Type Tissue Culture Collection (ATCC, Rockville, MD), were cultured at 37°C in a humidified atmosphere of 5% CO_2_. Cells were grown in Dulbecco's modified Eagle's medium (DMEM) supplemented with 8% fetal bovine serum (FBS).

### Western blot analysis

Cells cultured under various conditions were washed with PBS. After washing, cells were lysed with sodium dodecyl sulphate (SDS) sample buffer (10 mmol/L Tris-HCl, 2% SDS, 50 mmol/L dithiothreitol, 2 mmol/L EDTA, 0.02% bromophenol blue, 6% glycerol, pH 6.8) and treated at 100°C for five min. Samples were separated by SDS-polyacrylamide gel electrophoresis (PAGE). Proteins were transferred to PVDF membranes (Immobilon, Millipore), blocked in 5% milk in PBS containing 0.05% Tween 20, incubated with primary antibodies and detected by horseradish peroxidase-conjugated secondary antibodies (Dako, Carpenteria, CA). In some experiments, the primary antibodies were diluted with Can-Get-Signal Solution 1 (TOYOBO, Osaka, Japan) to enhance antibody-antigen binding.

### Immunoprecipitation

Cells were lysed in RIPA buffer containing 40 mM Tris-HCl (pH 8,0), 100 mM NaCl, 1 mM EDTA, 1 mM DTT, 1% NP-40, 0.1% SDS, 0.1% sodium deoxycholate supplemented with Complete™ protease inhibitor cocktail (Roche) and PhosSTOP phosphatase inhibitor cocktail (Roche Molecular Biochemicals, Indianapolis, Indiana). Lysates were cleared by centrifugation at 12,000× g for ten min, followed by immunoprecipitation using the indicated antibodies. In some experiments, RNase (10 µg/mL, Sigma) was added to the RIPA buffer to reduce non-specific binding of proteins to the protein A/G beads. Samples were separated by SDS- PAGE and analysed by Western blot analyses.

### RNA interference

Target sequences for short hairpin RNA (shRNA)-mediated knock-down of Girdin and Par-3 were described previously [6], [7], [8], [47]. A set of single-stranded oligonucleotides encoding the Girdin/Par-3 target sequences and their complements were synthesized as follows (only the sense sequence is shown): human Girdin shRNA (sh1), 5′-GGAACAAACAAGATTAGAA-3′ (nucleotides 3837–3855); human Girdin shRNA (sh7), 5′- GAAGGAGAGGCAACTGGAT -3′ (nucleotides 4166–4184); and human Par-3 shRNA (#4), 5′-GTGAAATTGAGGTCACACC-3′ (nucleotides 368–386). The oligonucleotide pair was annealed, inserted into the pSIREN-RetroQ retroviral shRNA expression vector (Clontech, Palo Alto, CA). To produce retroviral supernatants, GP2-293 packaging cells were seeded in collagen type I-coated 100 mm cell culture dishes and transfected with the pVSV-G (vesicular stomatitis virus G protein) vector and either control or Girdin/Par-3 shRNA-containing pSIREN-RetroQ vector using Lipofectamine 2000 reagent (Invitrogen). The medium was replaced 24 hrs later, and virus-containing supernatants were harvested 48 hrs post-transfection and used for following infection to Vero and MCF10A cells.

### Fluorescent immunocytochemistry

Vero cells, grown on poly-d-lysine (PDL)-coated glass base dishes, were fixed in 4% (w/v) paraformaldehyde, permeabilized with PBS containing 0.05% (v/v) TritonX-100 and then incubated with antibodies, followed by staining with Alexa 488/594-conjugated goat anti-mouse or anti-rabbit IgG (Invitrogen). After washing in PBS, fluorescence was visualized with a confocal laser scanning microscope Fluoview FV500 (Olympus).

### In vitro wound healing assay

Directional cell migration of Vero cells was stimulated in a monolayer by using an *in vitro* scratch wound assay. Vero cells were infected with retroviruses expressing the indicated shRNAs, and seeded on collagen type I-precoated 35 mm glass dishes. Confluent Vero cells were scratched with a 200 µL disposable plastic pipette tip and were allowed to migrate toward the wound. After wounding the cell monolayers, the medium was replaced with DMEM supplemented with 3% FBS. The cells were fixed at the indicated times for immunofluorescent staining. Wound widths were recorded at zero, seven, and nine hr after wounding. Data are presented as means ± S.E. Statistical significance was evaluated with Student's *t* test.

### Time-lapse recording of migrating cells

Vero cells were seeded on collagen type I-coated glass-based dishes and were placed in a CO_2_ incubator equipped with the LCV110 incubator fluorescence microscope (Olympus). After a 30 min equilibration period, differential interference contrast (DIC) images were obtained every four min for 16 hr with a cooled CCD camera controlled by MetaMorph software (ver. 7.6, Molecular Devices Corporation, Sunnyvale, CA). Stacks of images were analyzed by the ImageJ software (National Institutes of Health, Bethesda, MD) with the Manual Tracking plug-in for analysis of the track and velocity of each moving cell. The velocities of migrating cells (60 and 46 cells for the control and the Girdin deletion group, respectively) were determined by tracking the positions of cell nuclei using ImageJ software.

### Three-dimensional morphogenesis assay

Human mammary epithelial cells (MCF10A) obtained from the ATCC were cultured in DMEM/F12 (Invitrogen) supplemented with 5% horse serum (Invitrogen), 20 ng/mL EGF (Peprotech, Rocky Hill, NJ), 0.5 µg/mL hydrocortisone (Sigma), 100 ng/mL cholera toxin (Wako, Japan), and 10 µg/mL insulin (Sigma) in a humidified 37°C incubator with 5% CO_2_. Populations of MCF10A cells stably expressing control shRNA, Girdin shRNA or Par-3 shRNA were generated by retroviral infection and used for acinar morphogenesis assays as previously described elsewhere with some modifications [54], [55], [56], [63]. MCF-10A stable cell lines were trypsinized and suspended at a concentration of 10^5^ single cells per four mL. The cells were mixed 1∶1 with assay medium (DMEM/F12 supplemented with 2% horse serum, 10 ng/mL EGF, 5% Matrigel, 0.5 µg/mL hydrocortisone, 100 ng/mL cholera toxin, and 10 µg/mL insulin) and added to each chamber of a 24-well Matrigel invasion chamber (BD Biosciences, San Jose, CA, 5000 cells/400 µL/well). Assay medium containing 5 ng/mL EGF and 2.5% Matrigel was replaced every four days. Day 15 acini structures were fixed, stained for the respective antibodies, mounted, and visualized with a confocal laser scanning microscope.

### Data analysis

Data are presented as the means ± S.E. Statistical significance was evaluated with Student's t test.
